# Can ancient and modern stressors be distinguished? A mixed-methods exploration of psychosocial characteristics and health symptoms in young and older adults

**DOI:** 10.1177/1359105320965654

**Published:** 2020-10-15

**Authors:** Evangelos Katsampouris, Julie M Turner-Cobb, Julie C Barnett, Rachel S Arnold

**Affiliations:** 1 Department of Psychology, University of Bath, UK; 2 Department of Psychology, Bournemouth University, UK; 3 Department for Health, University of Bath, UK

**Keywords:** ancient, emotions, health, modern, stress

## Abstract

A novel conceptualisation of stress includes a distinction between ancient (AS) and modern stressors (MS); the notion that established adaptive psychophysiological coping processes may enable individuals to better withstand AS than MS. Two consecutive mixed-methods studies assessed the feasibility of distinguishing between AS and MS in young and older adults, using questionnaires and interviews. MS were positively associated with cold symptoms in older adults; and five psychosocial characteristics were identified to profile AS and MS along a continuum. An evolutionary distinction between AS and MS provides an important psychological dimension in better understanding and assessing stress-health processes.

## Introduction

Stress is a complex concept consisting of psychosocial life event stressors that require people’s mental and physical adaptation and which in turn can affect health (Hughes et al., 2018; Slavich, 2019). Despite an increasing amount of work concerning the psychophysiological underpinnings of stress and its links to health (Segerstrom and O’Connor, 2012), relatively little focus has been given to the evolutionary concepts surrounding stress and their importance in its conceptualisation, measurement, and impact on health.

### Ancient and modern stressors, and links to emotion and health

Psychological stress, defined as ‘a particular relationship between the person and the environment that is appraised by the person as taxing or exceeding his or her resources and endangering his or her well-being’ (Lazarus and Folkman, 1984: 19), has been substantially advanced by the adaptive theory of allostasis, the idea that physiological adaptation to environmental conditions encountered by an individual, promotes change and enables a person to adapt to and cope with life stressors (Goldstein and McEwen, 2002; Sterling and Eyer, 1988). A social evolutionary perspective may contribute to better understanding of different stress mechanisms that develop and adapt over time in response to stressors. Whilst stress response mechanisms once enabled survival from environmental threats, modern day stressors may fail to serve their original function and instead result in ill-health (Bourdon et al., 2020). An implied distinction exists in conceptualising stress from an evolutionary perspective and its consequent effects on health.

Schreier and Evans (2003: 306) defined ancient stressors as ‘chronic demands that have likely accompanied the human species throughout its existence’, and modern stressors as ‘new demands originating during the Neolithic period, generally defined by the advent of agriculture 10,000-12,000 years ago’. This is the notion that established adaptive psychophysiological coping mechanisms enable individuals to cope with ancient environmental stressors, which have accompanied human kind from its beginning (e.g. close family members have had serious arguments with each other, a child has had to deal with people whose behaviour was frightening), rather than with modern demands (e.g. commuting to work, being hospitalised for a serious illness, a close family member being away from home a lot, parents losing their job or being unemployed). Schreier and Evans (2003) found that children exposed to more modern stressors, rather than to ancient stressors, were significantly and positively likely to show greater levels of hypothalamic-pituitary-adrenal (HPA) axis activity, concluding that this effect of ancient and modern stressors cannot be ascribed to stressor severity. They found evidence for differences in concentration of cortisol (overnight urinary) between ancient and modern stressors experienced in boys and girls, aged 8 to 10 years, and called for this ancient and modern stressor concept to be extended to assessment in adults (Schreier and Evans, 2003). Currently, no measure exists to specifically assess ancient and modern stressors. However, an ancient/modern stressor classification adds a meaningful dimension to stress assessment, since it moves away from the association between perceived stressor severity and health and introduces a psychological distinction based on the origin of stressors that link to physical health outcomes.

To distinguish between ancient and modern stressors based on evolutionary concepts, self-conscious emotion (SCE) (e.g. shame, guilt), characterised by self-representations, self-evaluation and underlying evolutionary processes, is of particular relevance (Folger et al., 2014; Tangney and Dearing, 2003). Since SCEs are distinguishable by their adaptive coping profile (Luyten et al., 2002) ancient and modern stressors might also be distinguished by their adaptive/maladaptive coping status. As people are likely to be more able to cope with guilt, seen as a more adaptive moral emotion, rather than shame (Luyten et al., 2002), they might also be more able to cope with ancient rather than modern stressors due to established adaptive coping processes (Schreier and Evans, 2003). Inability to control and cope with stressful events elicits shame and guilt (Tracy and Robins, 2006), and the impact of SCEs on health has been considered (Dolezal and Lyons, 2017; Turner-Cobb et al., 2015). For example, psychosocial stressors associated with negative emotions and less effective coping have been positively associated with increased vulnerability and susceptibility to the common cold (Cohen et al., 2003). Thus, an ancient/modern stressor distinction might enable a better understanding of how stress can have differential effects on emotional, physiological and health outcomes.

### Objectives

This study assessed the feasibility of distinguishing between ancient and modern stressors in adults in relation to known associations between psychological stress and health outcomes. It aimed to examine the subjective experience of stress in two consecutively studied cohorts, one composed of young adults and the other of older adults, to assess evidence of underlying psychosocial characteristics sufficient to distinguish between ancient and modern stressors. It was hypothesised that compared to ancient stressors, modern stressors would reveal a stronger association with negative SCEs, poorer coping ability and health, in young and older adults.

## Study 1: Method

Data was collected from young adults between February and June 2015 using a sequential explanatory mixed-methods design with two phases (self-report questionnaires in phase 1; semi-structured interviews in phase 2). Full ethical approval was granted for study 1 by the University Departmental Ethics Committee (#15-006).

### Participants

Ninety-eight young adults (*M* = 20.33 years, *SD* = 1.74, range: 18–24 years) (60 females) were recruited using snowball sampling via social media and word-of-mouth, across the University for phase 1. The majority were white (80%), single (68%) and studying for an undergraduate science degree (85%). In phase 2, 40% of the initial cohort were re-contacted using purposeful sampling, drawn from the highest and lowest quintiles of the life events questionnaire scores (total number of life events), to obtain a sufficient sample with a range of responses, accounting for an attrition rate of 50%. Twenty participants (11 females) (*M* = 21.20 years, *SD* = 2.04) consented and participated in the in-person interviews (*M*length = 43.00 minutes, *SD* = 13.32, range: 30–80 minutes), which took place in the Department of Psychology laboratories.

### Measures

In phase 1, young adults were provided with a set of self-report questionnaires to assess psychological factors of relevance to the classification of ancient and modern stressors.

Life events were assessed using the Life Events Inventory (LEI) measuring the life change or the emotional distress level an event caused in the last year. The 67-item LEI generally refers to chronic stress and involves a wide range of desirable or undesirable life events experienced by individuals in everyday life, which can be considered as stressful and negative (Tennant and Andrews, 1976). Participants were asked to read each statement and to indicate how stressful the event had been for them, if it had happened in the last year. Participants scored severity (1: not at all stressful − 7: extremely stressful) as a way of indicating the life event had happened. LEI was rated on an 8-point Likert scale; 0 (*not happened*), and from 1 (*happened; not at all stressful*) to 7 (*happened; extremely stressful*); for this sample, Cronbach’s α = .93. The total number and mean severity score of life events were computed for each participant.

Perceived stress was assessed using the 10-item Perceived Stress Scale (PSS-10) measuring ‘the degree to which situations in one’s life are appraised as stressful’, ‘unpredictable, uncontrollable, and overloading’ in the past month (Cohen et al., 1983: 385–387). PSS-10 was scored on a 5-point Likert scale: 0 (*never*) to 4 (*very often*); Cronbach’s α = .78.

Self-conscious emotions (SCEs) were assessed using the Test Of Self-Conscious Affect (TOSCA-3) (Tangney and Dearing, 2003) composed of 11 negative and five positive scenarios assessing shame-proneness, guilt-proneness, externalisation, pride and detachment/unconcern. Participants were asked to imagine themselves in each scenario and to indicate how likely they would react in each one from 1 (*not likely*) to 5 (*very likely*) in a 5-point Likert scale; Cronbach’s α ⩾ .70.

Ancient and modern stressors: this was derived from the life event items from the LEI which was matched by meaning against the empirical-based a priori 32-item ancient and modern stressor designation list by Schreier and Evans (2003) (see Table 1; Supplemental Material). Four members of the research team independently performed this matching process; allocations were discussed until agreement was reached. Nine life events (five ancient and four modern stressors as designated in the original list) emerged as the most relevant and consistently agreed upon. Death/bereavement, movement, health/illness of others, and social/interpersonal arguments were designated as ancient stressors. Unemployment, financial problems, health/illness of self, and separation/distance were designated as modern stressors (see Table 1; Supplemental Material). The total number and mean severity score of ancient and modern stressors were computed for each participant.

### Interviews

During phase 2 of the study, one-on-one semi-structured in-person interviews were conducted and audio-recorded. An interview protocol was used (see Supplemental Material) which was based around the topics covered in phase 1, specific to each participant and reflecting their questionnaire responses. Interviewed participants were asked about their experiences, thoughts, and feelings of stressful life events reported, and how they coped with stressors and SCE responses.

### Procedure

Participants were given detailed information about the study through a participant information sheet prior to obtaining written consent at both phases. They were asked to attend an in-person 30-minute laboratory appointment with the researcher to complete questionnaires. Participants were informed that they would be re-contacted, if selected, based on the selection criteria described above, to participate in phase 2. Selected participants were asked to attend a 60 to 80 minute in-person interview. Although the study did not employ deception, a debrief sheet was provided at completion of the interview to explain the concept of ancient and modern stressors.

### Analytical plan

For phase 1 data, preliminary correlational analyses were conducted to identify associations between the variables of interest, followed by inferential statistics in IBM SPSS Statistics software v.22 using multiple regression to examine associations between the independent variables of ancient and modern stressors (total number; mean severity) and gender, and the dependent variables of SCEs. The designation of ancient and modern stressors was facilitated by use of a questionnaire assessment followed by interviews through which subjective reflection on the stress experience provided information relevant to underlying stressor characteristics to enable us to designate ancient and modern stressors. This designation was informed by the initial Schreier and Evans (2003) categorisation but substantially revised and developed by exploring the underlying stressor characteristics along a continuum.

For phase 2, interviews were transcribed verbatim and coded by hand. A deductive, directed, latent qualitative content analysis (CA) was conducted. The description of each life event stressor matched from the questionnaires was thoroughly examined to identify specific psychosocial characteristics that distinguished between ancient and modern stressors. The stages of CA were: data preparation; identification of category definitions from theory; reading and annotations of interview transcripts; decontextualisation, data coding and categorisation; data recontextualisation and review; compilation (explicit definitions and theory-driven coding rules for each category); and a report (Bengtsson, 2016). Regarding the second stage, the categories and definitions (see Table 1) were drawn from published work on characteristics of psychosocial stress (e.g. Anisman and Merali, 1999; Lazarus and Folkman, 1984; Schneiderman et al., 2005). An adult male researcher asked participants questions about a range of life stress experiences and their emotions and maintained a sympathetic but neutral tone throughout the interviews. An interview protocol mainly consisted of open-ended questions was used and participants were granted some space and time to discuss life stress experiences. The qualitative analysis was conducted by the first author and was also assessed, cross-checked and thoroughly discussed by the three other authors as members of the research supervisory group. Table 1 defines the ancient and modern stressor continuum across its polar extremes for the psychosocial characteristics of coping, experience, manageability/expectedness, duration and type.

**Table 1.** Definitions of psychosocial stressor characteristics categories.

**Adaptive:** Quotes which indicate or imply any form of adequate, available, functional, constructive, protective, appropriate, effective or successful coping strategies to deal with stress (e.g. problem-focused, emotion-focused, meaning-based coping). The outcome of adaptive coping can be favourable; quotes of a favourable outcome illustrate a resolved stressful event.	**←**	**Coping**	**→**	**Maladaptive:** Quotes which indicate or imply non-coping, problematic, less adaptive, dysfunctional, unsuccessful, less effective coping strategies to deal with stress. The individual would be less able and need more time to adapt and deal efficiently with a stressful event. The outcome of maladaptive coping can be unfavourable; quotes of an unfavourable outcome illustrate an unresolved stressful event.
**Past:** Quotes which indicate or imply that the individual has encountered and experienced a stressful event before. Based on an evolutionary perspective, a stressor that has been an integral part of the human experience from the beginning can be regarded as innate to the individual (e.g. bereavement).	**←**	**Experience**	**→**	**Novel:** Quotes which indicate or imply any form of uncertainty, unfamiliarity or dishabituation to a stressful event. Based on an evolutionary perspective, a stressor that has been more recent to the human experience because of modern life can be regarded as more modern to the individual (e.g. unemployment).
**Controllability/Predictability:** Quotes which indicate or imply predictability or control over the stressor; the stressor could potentially or was expected to occur.	**←**	**Manageability/Expectedness**	**→**	**Uncontrollability/Unpredictability:** Quotes which indicate or imply unpredictability or lack of control over the stressor because the stressor was not expected to occur.
**Short:** Quotes which indicate or imply that the stressful event lasted for a short period of time or had intermittent frequency.	**←**	**Duration**	**→**	**Long:** Quotes which indicate or imply that the stressful event lasted or was sustained for a long period of time or had continuous frequency. Time pressure has been considered as an underlying factor which affected long-lasting stressors.
**Simple:** Quotes which indicate or imply that the individual encountered and experienced a specific stressful event, which had not been affected by other several stressors.	**←**	**Type**	**→**	**Complex/Multiple:** Quotes which indicate or imply that the individual encountered and experienced a series of stressors. The whole stressful situation was more complex and multiple because of the presence of other several stressful factors, which escalated the experience of the stressor.
**↓**				**↓**
**Ancient**	**←**	**Stressor continuum**	**→**	**Modern**

To ensure rigor, reliability and trustworthiness of stressor designation, an inter-rater reliability test was conducted. Four researchers independently categorised a sample of randomly selected quotes into the stressor characteristic categories. The overall coding consensus criterion showed a high inter-rater reliability agreement for the young adults, κ = .81, *p* = 0.001, 95% CI [.653, .973] (Fleiss et al., 2003), according with the a priori designation by Schreier and Evans (2003). Triangulation, which enabled the use of two methods to gather data and examine ancient and modern stressors, involved the application of universal criteria (worthy topic, rich rigor, sincerity, credibility, resonance, significant contribution, ethics, meaningful coherence) (Tracy, 2010).

## Study 1: Results

### Descriptive and preliminary analyses

Mean scores are indicated for ancient and modern stressors and SCEs (see Table 2; Supplemental Material). The more ancient and modern stressors that the young adults reported, the lower were the levels of reported shame (see Table 3; Supplemental Material).

### Main effects

The total number of ancient and modern stressors and gender predicted shame (*R*^2^ = .22, *F*(3, 94) = 8.85, *p* < 0.001) in the overall regression model; the total number of ancient stressors (*p* = 0.002) and gender (females) (*p* = 0.011) were significant individual predictors (see Table 2).

**Table 2.** Summary of multiple regression analyses for predictor variables on shame in young adults (*N* = 98).

Predictors	Shame^a^ (*R* = 0.469)			
	*B* (*SE*)	β	*t* (*df*)	95% CI
Total number Ancient stressors	−0.116 (0.036)	−0.344	−3.20 (3, 94)**	−0.188, −0.044
Total number Modern stressors	−0.018 (0.060)	−0.033	−0.304 (3, 94)	−0.137, 0.100
Gender	0.295 (0.113)	0.243	2.61 (3, 94)*	0.071, 0.520

a Shame in the young adult population was assessed using the TOSCA-3.

* *p* ⩽ 0.05, **p ⩽ 0.01, ***p ⩽ 0.001.

### Content analysis

Five psychosocial characteristics emerged through CA that were deemed to underlie a distinction between ancient and modern stressors in young adults: (i) coping; (ii) experience; (iii) manageability/expectedness; (iv) stressor duration; and (v) stressor type. For ancient stressors (e.g. close family members have had serious arguments with each other, a child has had to deal with people whose behaviour was frightening), these were adaptive coping, past experience, controllability/predictability, short duration, and a simple stressor. For modern stressors (e.g. commuting to work, being hospitalised for a serious illness, a close family member being away from home a lot, parents losing their job or being unemployed), these were maladaptive coping, novel experience, uncontrollability/unpredictability, long duration, complex and multiple stressors. Death/bereavement, movement, health/illness of others and social/interpersonal arguments were designated as ancient stressors. Unemployment, financial problems, health/illness of self and separation/distance were designated as modern stressors (see Table 1; Supplemental Material).

Coding of ancient stressors indicated that young adults could adapt more easily than for modern stressors; adaptive coping resulted in favourable outcomes. Ancient stressors were not considered as evolutionarily novel as they had been experienced previously; could be controlled and expected to occur; typically lasted for a short time; and were frequently experienced as a specific single situation. Young adults were less able to deal with modern stressors (e.g. ‘That’s the idea of that actually destroyed me, I cannot see myself doing whatever I am going to do without sports like during the week . . . . That event was quite stressful because I lost a part of myself at that point. I cannot deal with it, I have to wait. (15/Participant 4)’; ‘In terms of having enough money to pay my loan, to pay my rent, so that’s very stressful because sometimes I don’t have any solutions so then I am getting upset and I have to ask people for help and I don’t want to do this which makes me thinking of the future and pushing me under pressure. (21/Participant 9)’); maladaptive coping likely to result in unfavourable resolutions. Modern stressors could be regarded as novel because individuals faced uncertainty and unfamiliarity; could not be typically controlled and predicted that it might occur; lasted or were sustained for a long time or had continuous frequency; and were experienced as a series of several multiple events resulting in the stressful situation being holistically more complex.

## Study 2: Method

Data was collected from older adults between April and October 2016 using the same sequential explanatory mixed-methods design as described for study 1. Full ethical approval was granted for study 2 by the University Departmental Ethics Committee (#16-057).

### Participants

Seventy-five older adults (*M* = 68.95 years, *SD* = 5.30, range: 60–75 years) (44 females) were recruited across the local county, in the same way as in study 1. The majority were white (100%), married (73%), retired (64%) and had obtained an undergraduate degree or above (21%). Forty older adults were approached for phase 2 and of those 21 participants (14 females) (*M* = 68.33 years, *SD* = 4.82) consented. As for study 1, interviews (*M*length = 54.05 minutes, *SD* = 22.28, range: 30–90 minutes) took place in the Department of Psychol-ogy laboratories.

### Measures

Participants were provided with a set of self-report questionnaires, made up of two of the three measures used in study 1, the Life Events Inventory and the Perceived Stress Scale (Cronbach’s α = .85 and α = .78, respectively for study 2). Self-conscious emotions focused specifically on shame and guilt, and in addition a health assessment of the occurrence of the common cold was included.

Shame was assessed using the Internalized Shame Scale (ISS) (Cook, 1988) measuring the frequency of shame statements experienced with which individuals may be familiar. ISS was scored on a 5-point Likert scale: 0 (*never*) to 4 (*almost always*); Cronbach’s α ≈ .95. Guilt was assessed using the Guilt Inventory (GI) (Jones et al., 2000) measuring trait and state guilt, and general moral standards. GI was scored on a 5-point Likert scale: 1 (*strongly agree*) to 5 (*strongly disagree*); Cronbach’s α = .79–.89. Different SCE measures were used in study 2 because significant associations were found between life events and shame, and guilt in study 1; and shame and guilt have been regarded as the most distinct SCE experiences (Tangney and Dearing, 2003).

Common cold symptoms were assessed using the Wisconsin Upper Respiratory Symptom Survey (WURSS-21) (Barrett et al., 2009) assessing the negative impact of cold symptoms on quality of life. WURSS-21 was scored on a 4-point Likert scale: 1 (*none*) to 4 (*severe*); Cronbach’s α = .76–.96.

Ancient and modern stressors were derived in the same way as for study 1.

### Interviews

One-on-one semi-structured in-person interviews were conducted and audio-recorded using a tailored interview protocol as for study 1 (see Supplemental Material) with the addition of questions about life events and common cold symptoms experienced.

### Procedure

Consenting procedures, phase 1 questionnaire assessment, phase 2 interview, and debriefing procedures used were the same as for study 1. The only differences in the procedure of study 2 compared to study 1 were firstly that some older adults returned their questionnaires via the mail rather than visiting the laboratory, and secondly three participants were interviewed over the phone.

### Analytical plan

The same analytical plan was followed as for study 1, using preliminary correlational analyses followed by multiple regression with the addition of the common cold symptoms (total number; mean severity) as a dependent variable. The ancient and modern stressor continuum and its psychological characteristics (see Table 1) were applied to this older adult cohort using the same methods as for study 1. The overall coding consensus criterion showed a high inter-rater reliability agreement for these older adults, κ = .83, *p* < 0.001, 95% CI [.700, .961] (Fleiss et al., 2003).

## Study 2: Results

### Descriptive and preliminary analyses

Mean scores are indicated for ancient and modern stressors, shame and guilt, and common cold symptoms (see Table 2; Supplemental Material). The more modern stressors the older adults reported, the higher the numbers of reported shame and cold symptoms (see Table 3; Supplemental Material).

### Main effects

For older adults, the total number of ancient and modern stressors and gender predicted shame (*R*^2^ = .28, *F*(3, 71) = 9.10, *p* < 0.001) in the overall regression model; the total number of modern stressors (*p* = 0.018) and gender (females) (*p* = 0.001) were significant individual predictors. Mean severity of ancient and modern stressors predicted guilt (*R*^2^ = .11, *F*(2, 72) = 4.32, *p* = 0.017); mean severity of ancient stressors was a significant independent predictor (*p* = 0.010). Mean severity of ancient and modern stressors predicted cold symptoms in the overall model (*R*^2^ = .12, *F*(2, 72) = 5.10, *p* = 0.008); mean severity of modern stressors was a significant individual predictor (*p* = 0.010) (see Table 3).

**Table 3.** Summary of multiple regression analyses for predictor variables shame, guilt and common cold symptoms in older adults (*N* = 75).

Predictors	*B* (*SE*)	β	*t*(*df*)	95% CI
	Shame^a^ (*R* = 0.527)			
Total number Ancient stressors^b^	0.036 (0.061)	0.070	−	−0.069, 0.165
Total number Modern stressors^b^	0.327* (0.149)	0.300	−	−0.072, 0.535
Gender	0.628*** (0.139)	0.413	−	0.347, 0.891
	Guilt^c^ (*R* = 0.327)			
Mean severity Ancient stressors^b^	−0.061** (0.022)	−0.336	−	−0.106, −0.019
Mean severity Modern stressors^b^	0.006 (0.022)	0.032	−	−0.037, 0.052
	Common cold symptoms (*R* = 0.352)			
Mean severity Ancient stressors^b^	0.041 (0.070)	0.072	−	−0.098, 0.162
Mean severity Modern stressors^b^	0.193*** (0.071)	0.323	−	0.063, 0.333

a Shame in the older adult population was assessed using the ISS.

b Bootstrapped; Gender, coded: 1 = Male, 2 = Female.

c Guilt in the older adult population was assessed using the GI.

* *p* ⩽ 0.05, **p ⩽ 0.01, ***p ⩽ 0.001.

### Content analysis

The same life event stressors were assessed (see Table 1; Supplemental Material) and the same five psychosocial characteristics emerged through CA as observed for study 1 (see Table 1). These characteristics were deemed to underlie a distinction between ancient and modern stressors in this older adult sample: (i) coping; (ii) experience; (iii) manageability/expectedness; (iv) stressor duration; and (v) stressor type. The data that was collected from older adults were analysed independently and separately from the data collected for young adults. The findings in study 2 were identical and echoed that of study 1. Although older adults experienced modern stressors as more stressful than ancient stressors because of the lack of adaptive coping mechanisms, they reported during the interviews that they experienced modern stressors as less stressful than did young adults. Coping skills were reported by older adults as acquired over the lifecourse and past experiences were cited as useful in dealing with stressors designated as ancient.

## Discussion

This study set out to assess whether it was feasible to distinguish between ancient and modern stressors in adults, and to evaluate whether stress appraisals by young and older adults might provide evidence to support the delineation of underlying stressor characteristics relevant to ancient and modern stressors. Overall, support was found for the classification of stressors as ancient or modern, with evidence from young and older adults revealing associations between ancient/modern stressors, psychological variables and health. However, findings suggest that this concept is viewed along a continuum as opposed to a simple dichotomy. It was hypothesised that modern stressors would reveal a psychosocial profile of stronger association with negative SCEs, poorer coping ability and health in young and older adults. Partial support was provided for this hypothesis.

Quantitative findings in young adults, provided evidence for shame being significantly associated with total number of ancient stressors, and women reported greater levels of shame than men. In older adults, modern stressors were significantly associated with shame, and women reported greater levels of shame than men. Modern stressors were significantly associated with common cold symptoms, and ancient stressors were a significant predictor of guilt. The theoretical implications of these findings accord with the transactional theory of stress and coping, theories of adaptation and SCEs (Lazarus and Folkman, 1984; Sterling and Eyer, 1988; Tangney and Dearing, 2003). For example, individuals reported shame and guilt associated with stressful psychosocial events that were appraised as being important to well-being and self-evaluation, and which they found it difficult to cope with, as reported in previous work (Dickerson and Kemeny, 2004; Henry et al., 2016; Tracy and Robins, 2006). Ancient stressors might be appraised as important and elicit negative SCEs if people have not been able to cope with these stressors. The interpretation of this finding is difficult in the absence of literature linking ancient/modern stressors with shame/guilt. There is limited evidence about gender differences in SCEs regarding stressful situations, except for evidence in self-stereotyping-related situations (Else-Quest et al., 2012).

In older adults, modern stressors were found to be significantly associated with cold symptoms while there was no main effect of overall perceived stress on symptoms. Common cold symptoms were used as an illness paradigm in the second study with older adults in order to assess links between ancient and modern stressors with health outcomes. Compared to ancient stressors, individuals might be less able to adapt and cope with modern stressors and may experience greater and prolonged physiological arousal resulting in allostatic load and ill-health (McEwen, 2006; Sterling and Eyer, 1988). Older adults, who experience modern stressors, might be more likely to be susceptible and vulnerable to physical health symptoms (Cohen et al., 2003; Glei et al., 2007), either because of their inability to cope or because modern stressors impact allostatic systems to a greater degree. Maladaptive coping has been regarded as a mediating pathway between stress exposure and negative health outcomes (Lazarus, 2000).

In the qualitative analysis, five psychosocial characteristics were identified as underlying ancient and modern stressors: coping, experience, manageability/expectedness, duration, and type of stressor. CA assessed only nine specific life event stressors, which were a priori empirically-designated as ancient and modern (Schreier and Evans, 2003), providing a plausible rationale to provisionally distinguish between these stressors. Findings suggest that ancient and modern stressors are viewed along a continuum rather than as a simple dichotomy; thus, stressors, irrespective of a person’s age and gender, can be perceived as ancient or modern depending upon the individual’s appraisals and adaptive/maladaptive coping responses and associated psychosocial characteristics (i.e. coping, experience, manageability/expectedness, duration, type). For example, in these current studies the life event of death/bereavement was mainly considered by some participants as an ancient stressor as they could cope with this life stressor and had experienced it before. Whilst other participants, who had experienced this life stressor for the first time, were also able to cope with it and considered this life event as an ancient stressor. This proposed continuum indicates that individuals’ experiences and coping responses, and stressor characteristics might constitute factors which determine why a life stressor could be regarded as more ancient or more modern by each individual. Stressors may have a different profile regarding individuals’ appraisals or differential effects on health-related outcomes. It is possible that modern rather than ancient stressors can be associated with poorer health outcomes in older adults, for whom the stressor distinction may be more accentuated.

Thus the theory and growing evidence suggests that people are evolutionarily capable of coping with stressors which have been around for many years rather than with more modern or recently evolved stressors (Schreier and Evans, 2003). The idea is that individuals are capable of adapting their behaviour through inherited hypo-egoic predispositions and coping with situations that ancestors used to deal with (Leary et al., 2006). This accords with the notion that, regardless of age, established adaptive psychophysiological coping processes enable people to innately cope with familiar ancient stressors which have been an integral part of human evolution (Schreier and Evans, 2003). Similarly, the fight-flight stress response might not be so suitable for coping with modern stressors in contemporary society, since modern stressors have become increasingly complex and might need a greater level of cognitive appraisal.

The inability to cope with unpredictable, novel and uncontrollable stressors is more likely to cause additional strain to people, increased cortisol responses and negative health outcomes (Dickerson and Kemeny, 2004). Stressor characteristics such as novelty, unpredictability, uncontrollability, maladaptive time perspectives, and threat to the self can result in activation of the HPA axis responses (Bourdon et al., 2020). Modern stressors were found to be profiled by such characteristics and multiplicity/complexity, as people might need more time to cope with stressors that have not previously been experienced or mismatch with the familiar ancient past (Li and Kanazawa, 2016). This incompatibility between ancient/non-industrial environments and modern life situations might result in maladaptive responses and poorer health (Flinn et al., 2011; Trevathan, 2007).

In the current studies, no gender differences were found in coping with ancient and modern stressors for young and older adults. This finding accords with Schreier and Evans (2003) suggesting that boys and girls did not differ in their ancient and modern stressor experiences. Male and female, young and older adults, were found to deal with ancient stressors using problem-focused, emotion-focused, and meaning-based adaptive coping strategies. Young and older adults experienced modern-designated stressors as more stressful than ancient stressors. In the current studies, regardless of gender and age, individuals coped with ancient stressors and did not indicate any form of adaptive coping with modern stressors. This is congruent with the notion that established adaptive psychophysiological coping processes enable people to cope with ancient stressors to a greater degree rather than with modern stressors (Schreier and Evans, 2003). No gender differences were found in SCE experiences regarding ancient and modern stressors for young and older adults in the current studies; and ancient/modern stressors were not distinguishable by their SCE adaptive/maladaptive coping profile.

A further concept, that of social-evaluative threat (SET) (Dickerson and Kemeny, 2004) was not directly assessed in the present study, but would be an important concept to consider in future research on ancient and modern stressors. Whether SET is an overarching factor in relation to the stressor characteristics of ancient and modern stressors, if it acts as a stressor characteristic per se, or if it is embedded within the stressor characteristics we have identified as core ancient/modern stressors, remains to be seen. The role of SET in the stress response would enable further in-depth understanding of this stressor distinction and examination of possible links with health outcomes as seen in other domains of stress research (Gruenewald et al., 2006). The current work represents the first attempt to provide empirical evidence of underlying psychosocial characteristics sufficient to explore the feasibility of distinguishing between ancient and modern stressors within a health context; and to potentially contribute to a novel and innovative conceptualisation of stress, its measurement, and impact on health via allostatic pathways.

There are a number of limitations in the studies presented. Participants were university students and older adults entirely recruited from the south-west region of the U.K. Future research would benefit from recruiting adults from several sociocultural backgrounds to further explore adults’ perceptions of ancient and modern stressors. Interviewed participants were re-contacted approximately 4 weeks after questionnaire completion and were not interviewed until a further 2 weeks later. Due to this delay, some participants might have experienced difficulty in remembering their questionnaire responses and in recalling memories and experiences. The relatively long interview duration might have resulted in participant burden and use of fewer topics might have enabled more time to focus on a specific topic of interest. The present study also faced some methodological complexities because of the novelty of the ancient/modern stressors and the lack of research around this concept, and the lack of clarity of methodological constructs that were employed in the original work by Schreier and Evans (2003). Future work would benefit from assessing ancient/modern stressors using experimental methods. The studies lacked bio-physiological measurement (e.g. endocrine assessment); future research is needed that includes an examination of the extent to which ancient and modern stressors might influence health outcomes at a biological level. According to the theory of allostasis, it would be expected that modern stressors would be positively associated with higher cortisol release and HPA axis activity in adults, resulting in poorer health implications. This physiological work would strengthen and validate the provisional stressor distinction. Importantly, there is currently no measure to assess ancient and modern stressors and development of such a standardised psychological measure would enable researchers to further this research and assess the evidence reported in the current studies in applications across different age groups and populations.

## Conclusion

This research was the first to explore from a psychological perspective the feasibility of distinguishing between ancient and modern stressors within a health context using an in-depth mixed-methods examination in young and older adults. It assessed the appraisal of psychosocial stressors and associations with SCE-proneness, coping, and common cold symptoms regarding ancient and modern stressors. Findings suggest some empirical evidence of psychosocial characteristics enabling a provisional ancient/modern stressor distinction; the notion that individuals may be better able to withstand ancient than modern stressors due to established adaptive psychophysiological coping processes. This research provides a better understanding of psychosocial stress and identified stressors that might have the most deleterious effect on health. Future research is called for to further develop the assessment and application of the ancient/modern stressor distinction in other contexts such as an experimental setting, moving beyond the explicit distinction that was found in this study and assessing the feasibility of an implicit distinction. Consideration of an ancient/modern stressor distinction is suggested as important in understanding the impact of the stress experience on health and it provides a valuable addition to the armoury of stress research.

## Supplemental Material

Supplementary_Material_-_JHP-20-0254.R1 – Supplemental material for Can ancient and modern stressors be distinguished? A mixed-methods exploration of psychosocial characteristics and health symptoms in young and older adults Click here for additional data file. Supplemental material, Supplementary_Material_-_JHP-20-0254.R1 for Can ancient and modern stressors be distinguished? A mixed-methods exploration of psychosocial characteristics and health symptoms in young and older adults by Evangelos Katsampouris, Julie M Turner-Cobb, Julie C Barnett and Rachel S Arnold in Journal of Health Psychology
