# Supplementary material for: Can ancient and modern stressors be distinguished? A mixed-methods exploration of psychosocial characteristics and health symptoms in young and older adults
Source: J Health Psychol. 2020 Oct 15;27(3):624–36. doi: 10.1177/1359105320965654 (PMC8832552; doi:10.1177/1359105320965654)
Supplement: Supplementary_Material_-_JHP-20-0254.R1 – Supplemental material for Can ancient and modern stressors be distinguished? A mixed-methods exploration of psychosocial characteristics and health symptoms in young and older adults [file Supplementary_Material_-_JHP-20-0254.R1.docx]

**Table 1**

Matching life event items based on original Schreier and Evans (2003) and Life Events Inventory used in present study

| **Schreier & Evans life events** | **Ancient/Modern** | **Life events inventory** | **Life events concept** |
| --- | --- | --- | --- |
| Another relative died with whom the child had a very close relationship. | Ancient | A close family friend or relative died (e.g., aunt, uncle, grandparent, cousin, etc.). | Death/bereavement |
| A parent lost his/her job or has been unemployed. | Modern | You have been unemployed and seeking work for a month or more. | Unemployment |
| A parent, brother, or sister died. | Ancient | A close family member died (e.g., partner, sibling, child, etc.). | Death/bereavement |
| Our family had serious financial problems. | Modern | You had a major financial crisis. | Financial problems |
| A close family member had a serious medical problem (illness or accident) and was in the hospital. | Modern | You had a serious illness, injury or operation needing hospitalisation, (or a month or more off University/work). | Health/illness of self |
| Our family has had to move a lot. | Ancient | You moved to Bath from the UK/Europe/overseas. You moved house in Bath. | Movement |
| A close family member was badly hurt or sick (but not in the hospital). | Ancient | A close relative had a serious illness (from which they did not die). | Health/illness of others |
| Our child was upset by family arguments. Our child has been involved in serious family arguments. Close family members have had serious arguments with each other. | Ancient | There has been increasing serious arguments with your partner. You had increasing arguments or difficulties with your partner (or steady friend). There has been a serious increase in arguments or problems with someone who lives at home (excluding your partner). | Social/interpersonal arguments |
| A close family member was away from home a lot. Our child’s best friend moved away. | Modern | You have been separated from your partner for more than a month because of personal difficulties. You have been separated from your partner for more than a month (for reasons other than relationship difficulties). You have been separated from someone important to you (other than close family members). | Separation/distance |

**Interview protocol – Study 1**

Participant’s no:

Interview date:

Time begun:

Time ended:

Duration of interview:

I am XXXXX and I work as a researcher at the University of XXXXX. I would like to remind you that the interview will be audio-recorded and I will be the only one who listens to it. I have got with me your questionnaire because I am going to ask you some questions in relation to the answers you gave on it. However, there are some things I need you to keep in mind throughout our discussions:

1) As a participant in this study you have several rights. Your participation is entirely voluntary and you are free to decline to answer any questions I will be asking or stop the discussions at any point. There are no right or wrong answers to the questions I will be asking. I want to learn and benefit from your experiences so that I can better understand the relationship between stress and emotions. Therefore, I hope that you will answer the questions in a candid and straightforward way. If there are any questions that you are not comfortable answering I would rather you decline to comment than tell me what you think I might want to hear. So if you would prefer not to answer a question, please simply state “No comment” and I will move straight onto the next question.

2) I will be asking you about your experiences and feelings on stressful life events that you referred to on the questionnaires. Answering the questions will likely involve you thinking back to events and incidents that have occurred a year or month ago. Since you will have to think back in time, you might not be able immediately remember some things. Take your time as you try to recall the past; pauses are fine. If you cannot remember after trying to think back, then just let me know, but please do not guess.

3) When you are doing this recall, keep in mind that I am interested in your overall experience. So, in your answers you can draw on any and all aspects of your experiences.

4) The interview contains several sections covering various aspects. I will sometimes use the term “stressor” during our discussions; the stressor refers to any life event which may have made you feel stressed. At the end of each section, there will be an opportunity for you to add anything else that you felt was important and not covered in the questions asked.

5) There are no significant disadvantages and risks of taking part in this study. The interviews will involve disclosure of stressful events and emotions that have happened to you. It is possible that talking about these events may trigger some upset depending on the meaning of those events for you. However, we are interested in everyday stresses encountered in day to day life rather than traumatic events. The researcher will be available whilst the informal interview is conducted.

Do you have any questions about what I have talked about so far? If you have any questions as we go along or if at any time you do not understand what I am asking and need some clarification, please just ask.

*Warm-up questions:*

Could you please tell me a bit about yourself?

What are you studying?

Why have you been interested in this study?

How have you found your course so far?

*Section 1:*

|  | Interview questions | Participant prompts |
| --- | --- | --- |
| 1.1 | Why was this life event stressful for you? | - Information - Meaning |
| 1.2 | When did it happen? | - Who was involved? - What triggered it? |
| 1.3 | Had you had any previous experience of this life event before or was it the first time it happened? | - If not, appraisal of the stressor? |
| 1.4 | How did you think/evaluate (appraise) this life event? | - Adaptation/   Maladaptation?   - Complexity/   Multiplicity of stressor?   - Lack of control? - Unpredictability? - Threat, loss, challenge, harm, stressful - Benign, irrelevant - Outcomes? - Severity? |
| 1.5 | How did you feel about this life event? | - Shame, guilt, embarrassment, pride, externalisation, blame, detachment - Why? |
| 1.6 | Before proceeding to the next section, is there anything else you can add concerning what has just been discussed in this section? |  |

*Section 2:*

|  | Interview questions | Participant prompts |
| --- | --- | --- |
| 2.1 | Could you adjust yourself (adapt) to this life event? | - Ability/Inability to adapt - Easy or difficult? - Ancient/Modern? - Why? |
| 2.2 | How did you deal with this life event? | - Ability/Inability to cope - Easy or difficult? - Effectiveness? - Ancient/Modern? - Why? |
| 2.3 | Is there anything else you can add concerning what has just been discussed in this section? |  |

*Closing questions:*

How do you think the interview went?

Did you feel you could tell your story fully?

Did I lead you or influence your responses in any way?

Is there anything that we have not talked about that you are able to tell me about your experiences?

Have you any comments or suggestions about the interview itself?

**Interview protocol – Study 2**

Participant’s no:

Interview date:

Time begun:

Time ended:

Duration of interview:

I am XXXXX and I work as a researcher at the University of XXXXX. I would like to remind you that the interview will be audio-recorded and I will be the only one who listens to it. I have got with me your questionnaire because I am going to ask you some questions in relation to the answers you gave on it. However, there are some things I need you to keep in mind throughout our discussions:

1) As a participant in this study you have several rights. Your participation is entirely voluntary and you are free to decline to answer any questions I will be asking or stop the discussions at any point. There are no right or wrong answers to the questions I will be asking. I want to learn and benefit from your experiences so that I can better understand the relationship between stress and emotions. Therefore, I hope that you will answer the questions in a candid and straightforward way. If there are any questions that you are not comfortable answering I would rather you decline to comment than tell me what you think I might want to hear. So if you would prefer not to answer a question, please simply state “No comment” and I will move straight onto the next question.

2) I will be asking you about your experiences and feelings on stressful life events that you referred to on the questionnaires. Answering the questions will likely involve you thinking back to events and incidents that have occurred a year or month ago. Since you will have to think back in time, you might not be able immediately remember some things. Take your time as you try to recall the past; pauses are fine. If you cannot remember after trying to think back, then just let me know, but please do not guess.

3) When you are doing this recall, keep in mind that I am interested in your overall experience. So, in your answers you can draw on any and all aspects of your experiences.

4) The interview contains several sections covering various aspects. I will sometimes use the term “stressor” during our discussions; the stressor refers to any life event which may have made you feel stressed. At the end of each section, there will be an opportunity for you to add anything else that you felt was important and not covered in the questions asked.

5) There are no significant disadvantages and risks of taking part in this study. The interviews will involve disclosure of stressful events and emotions that have happened to you. It is possible that talking about these events may trigger some upset depending on the meaning of those events for you. However, we are interested in everyday stresses encountered in day to day life rather than traumatic events. The researcher will be available whilst the informal interview is conducted.

Do you have any questions about what I have talked about so far? If you have any questions as we go along or if at any time you do not understand what I am asking and need some clarification, please just ask.

*Warm-up questions:*

Could you please tell me a bit about yourself?

Do you work or have you been retired?

Why have you been interested in this study?

*Section 1:*

|  | Interview questions | Participant prompts |
| --- | --- | --- |
| 1.1 | Why was this life event stressful for you? | - Information - Meaning |
| 1.1.1 | When did it happen? | - Who was involved? - What triggered it? |
| 1.2 | How did you feel about this life event? | - Shame, guilt; blame - Why? |
| 1.2.1 | Before proceeding to the next section, is there anything else you can add concerning what has just been discussed in this section? |  |

*Section 2:*

|  | Interview questions | Participant prompts |
| --- | --- | --- |
| 1.3 | How did you think/evaluate (appraise) this life event? | - Adaptation/   Maladaptation?   - Complexity/   Multiplicity of stressor?   - Lack of control? - Unpredictability? - Threat, loss, challenge, harm, stressful - Benign, irrelevant - Outcomes? - Severity? |
| 1.4 | How did you deal with this life event? | - Ability/Inability to cope - Easy or difficult? - Effectiveness? - Ancient/Modern? - Why? |
| 1.5 | Had you had any previous experience of this life event before or was it the first time it happened? | - If not, appraisal of the stressor? |
| 1.6 | Had this life event affected your health? | - Severity of stress on physical health? - Common cold symptoms? - Any ongoing chronic conditions? |
| 1.6.1 | Is there anything else you can add concerning what has just been discussed in this section? |  |

*Section 3:*

|  | Interview questions | Participant prompts |
| --- | --- | --- |
| 1.7 | How do you believe that you would think about and cope with this life event, if you experienced it in a younger age? | - Appraisal of stressor? - Adaptation and coping? - Ancient/Modern? |
| 1.7.1 | Is there anything else you can add concerning what has just been discussed in general? |  |

*Closing questions:*

How do you think the interview went?

Did you feel you could tell your story fully?

Did I lead you or influence your responses in any way?

Is there anything that we have not talked about that you are able to tell me about your experiences?

Have you any comments or suggestions about the interview itself?

**Table 2**

Means, standard deviations (*SD*) and range (minimum-maximum) of psychological variables and SCEs in young adults (*N* = 98), and common cold symptoms in older adults (*N* = 75)

| **Measures** | **Adults** | | | | | | | | |  |
| --- | --- | --- | --- | --- | --- | --- | --- | --- | --- | --- |
|  | **Young** | | | | **Older** | | | | |  |
|  | ***Mean*** | **(*SD*)** | **Min-** | **Max** | | ***Mean*** | **(*SD*)** | **Min-** | **Max** | |
| **Psychological variables** |  |  |  |  | |  |  |  |  | |
| Total number Ancient stressors | 2.88 | (1.78) | 0- | 8.00 | | 1.33 | (1.40) | 0- | 5.00 | |
| Mean severity Ancient stressors | 3.69 | (1.51) | 0- | 6.50 | | 2.37 | (2.18) | 0- | 6.25 | |
| Total number Modern stressors | .96 | (1.08) | 0- | 5.00 | | .35 | (.69) | 0- | 4.00 | |
| Mean severity Modern stressors | 2.17 | (2.17) | 0- | 7.00 | | 1.13 | (2.11) | 0- | 7.00 | |
| Perceived stress | 18.80 | (6.08) | 3.00- | 32.00 | | 14.65 | (7.52) | 0- | 36.00 | |
| **SCEs** |  |  |  |  | |  |  |  |  | |
| Shame | 2.78 | (.60)^a^ | 1.19- | 4.38^a^ | | .69 | (.75)^b^ | 0- | 2.89^b^ | |
| Guilt | 3.94 | (.41)^a^ | 2.50- | 4.81^a^ | | 3.14 | (.40)^c^ | 2.28- | 4.24^c^ | |
| Detachment | 2.76 | (.50) | 1.64- | 4.00 | | - | - | - | - | |
| Externalisation | 2.15 | (.47) | 1.06- | 3.50 | | - | - | - | - | |
| Alpha pride | 3.89 | (.62) | 2.20- | 5.00 | | - | - | - | - | |
| Beta pride | 3.99 | (.59) | 2.40- | 5.00 | | - | - | - | - | |
| Negative SCEs | 3.10 | (.30) | 2.26- | 3.69 | | - | - | - | - | |
| **Common cold symptoms** |  |  |  |  | |  |  |  |  | |
| Total number symptoms | - | - | - | - | | 3.89 | (4.90) | 0- | 15.00 | |
| Mean severity symptoms | - | - | - | - | | 1.26 | (1.26) | 0- | 3.29 | |

Note. ^a^ Shame and guilt in the young adult population were assessed using the TOSCA-3; ^b^ Shame in the older adult population was assessed using the ISS; ^c^ Guilt in the older adult population was assessed using the GI.

**Table 3**

Correlations between ancient and modern stressors and SCEs in young adults (*N* = 98), and common cold symptoms in older adults (*N* = 75)

| **Psychological variables** | **Adults** | | | | | | | |
| --- | --- | --- | --- | --- | --- | --- | --- | --- |
|  | **Young** | | | **Older** | | | | |
|  | **SCEs** | | **Gender** | **SCEs** | | **Gender** | **Common cold symptoms** | |
|  | **Shame**^a^ | **Guilt**^a^ |  | **Shame^b^** | **Guilt^c^** |  | **Total number** | **Mean severity** |
| Total number Ancient stressors | -.401*** | -.184 | -.162 | .183 | -.139 | .045 | .254* | .270* |
| Mean severity Ancient stressors | -.049 | .010 | .049 | .312** | -.336** | .170 | .185 | .172 |
| Total number Modern stressors | -.259** | -.159 | -.186 | .317** | -.137 | -.010 | .220 | .310** |
| Mean severity Modern stressors | -.152 | .073 | -.099 | .228* | -.072 | .060 | .261* | .346** |
| Gender | .305** | 356*** | - | .413*** | -.200 | - | .126 | .070 |
| Perceived stress | .321*** | .070 | .227* | .745*** | -.382*** | .312** | .203 | .281* |

Note. * *p* ≤ .05, ** *p* ≤ .01, *** *p* ≤ .001; Gender, coded: 1 = Male, 2 = Female; ^a^ Shame and guilt in the young adult population were assessed using the TOSCA-3; ^b^ Shame in the older adult population was assessed using the ISS; ^c^ Guilt in the older adult population was assessed using the GI.
